# Estradiol levels in women with hormone receptor-positive advanced breast cancer on fulvestrant therapy

**DOI:** 10.1093/oncolo/oyaf403

**Published:** 2025-12-05

**Authors:** Shuqin Dai, Yanling Wen, Xingping Wu, Xi Wang, Zhongyu Yuan, Fei Xu, Xiaoming Xie, Wen Xia, Shusen Wang, Yanxia Shi, Peng Liu, Qiufan Zheng, Jun Tang, Xin An, Jiajia Huang, Meiting Chen, Yongyi Zhong, Xiwen Bi, Yi Yang, Cong Xue

**Affiliations:** Department of Medicine Laboratory, State Key Laboratory of Oncology in South China, Guangdong Provincial Clinical Research Center for Cancer, Sun Yat-Sen University Cancer Center, Guangzhou 510060, P. R. China; Department of Medical Oncology, State Key Laboratory of Oncology in South China, Guangdong Provincial Clinical Research Center for Cancer, Sun Yat-Sen University Cancer Center, Guangzhou 510060, P. R. China; Department of Medicine Laboratory, State Key Laboratory of Oncology in South China, Guangdong Provincial Clinical Research Center for Cancer, Sun Yat-Sen University Cancer Center, Guangzhou 510060, P. R. China; Department of Breast Oncology, State Key Laboratory of Oncology in South China, Guangdong Provincial Clinical Research Center for Cancer, Sun Yat-Sen University Cancer Center, Guangzhou 510060, P. R. China; Department of Medical Oncology, State Key Laboratory of Oncology in South China, Guangdong Provincial Clinical Research Center for Cancer, Sun Yat-Sen University Cancer Center, Guangzhou 510060, P. R. China; Department of Medical Oncology, State Key Laboratory of Oncology in South China, Guangdong Provincial Clinical Research Center for Cancer, Sun Yat-Sen University Cancer Center, Guangzhou 510060, P. R. China; Department of Breast Oncology, State Key Laboratory of Oncology in South China, Guangdong Provincial Clinical Research Center for Cancer, Sun Yat-Sen University Cancer Center, Guangzhou 510060, P. R. China; Department of Medical Oncology, State Key Laboratory of Oncology in South China, Guangdong Provincial Clinical Research Center for Cancer, Sun Yat-Sen University Cancer Center, Guangzhou 510060, P. R. China; Department of Medical Oncology, State Key Laboratory of Oncology in South China, Guangdong Provincial Clinical Research Center for Cancer, Sun Yat-Sen University Cancer Center, Guangzhou 510060, P. R. China; Department of Medical Oncology, State Key Laboratory of Oncology in South China, Guangdong Provincial Clinical Research Center for Cancer, Sun Yat-Sen University Cancer Center, Guangzhou 510060, P. R. China; Department of Breast Oncology, State Key Laboratory of Oncology in South China, Guangdong Provincial Clinical Research Center for Cancer, Sun Yat-Sen University Cancer Center, Guangzhou 510060, P. R. China; Department of Medical Oncology, State Key Laboratory of Oncology in South China, Guangdong Provincial Clinical Research Center for Cancer, Sun Yat-Sen University Cancer Center, Guangzhou 510060, P. R. China; Department of Breast Oncology, State Key Laboratory of Oncology in South China, Guangdong Provincial Clinical Research Center for Cancer, Sun Yat-Sen University Cancer Center, Guangzhou 510060, P. R. China; Department of Medical Oncology, State Key Laboratory of Oncology in South China, Guangdong Provincial Clinical Research Center for Cancer, Sun Yat-Sen University Cancer Center, Guangzhou 510060, P. R. China; Department of Medical Oncology, State Key Laboratory of Oncology in South China, Guangdong Provincial Clinical Research Center for Cancer, Sun Yat-Sen University Cancer Center, Guangzhou 510060, P. R. China; Department of Medical Oncology, State Key Laboratory of Oncology in South China, Guangdong Provincial Clinical Research Center for Cancer, Sun Yat-Sen University Cancer Center, Guangzhou 510060, P. R. China; Department of Medical Oncology, State Key Laboratory of Oncology in South China, Guangdong Provincial Clinical Research Center for Cancer, Sun Yat-Sen University Cancer Center, Guangzhou 510060, P. R. China; Department of Medical Oncology, State Key Laboratory of Oncology in South China, Guangdong Provincial Clinical Research Center for Cancer, Sun Yat-Sen University Cancer Center, Guangzhou 510060, P. R. China; Department of Biochemistry, Zhongshan School of Medicine, Sun Yat-Sen University, Guangzhou 510080, P. R. China; Department of Medical Oncology, State Key Laboratory of Oncology in South China, Guangdong Provincial Clinical Research Center for Cancer, Sun Yat-Sen University Cancer Center, Guangzhou 510060, P. R. China

**Keywords:** estradiol monitoring, fulvestrant, gonadotropin-releasing hormone agonists, hormone receptor-positive advanced breast cancer, liquid chromatography–tandem mass spectrometry

## Abstract

**Background:** Limited data are available on estradiol (E2) levels during fulvestrant treatment in women with hormone receptor-positive breast cancer.

**Methods:** We measured plasma E2 levels in women receiving fulvestrant using liquid chromatography–tandem mass spectrometry. Patient characteristics and treatment efficacy were assessed in relation to E2 levels. A cutoff of 2.72 pg/mL was used because it defines E2 suppression and postmenopausal status.

**Results:** A total of 69 women were enrolled, with a median age of 48 years. The median duration of fulvestrant treatment was 11.6 months. The median E2 level across the cohort was 3.60 pg/mL, with considerable interindividual variability (range: 1.11-526.13 pg/mL), and 49 women (71.0%) had E2 levels above 2.72 pg/mL. Eleven women (15.9%) had premenopausal E2 levels (>10 pg/mL). During a median follow-up period of 8.4 months, there was no statistically significant difference in progression-free survival (PFS) between women with E2 levels >2.72 pg/mL and those with E2 levels ≤2.72 pg/mL (*P* = .391). However, among women who benefited from first- or second-line fulvestrant therapy (PFS > 6 months), those with E2 levels >2.72 pg/mL exhibited significantly poorer PFS compared to those with E2 levels ≤2.72 pg/mL (*P* = .043).

**Conclusions:** These findings support the need for E2 monitoring in women receiving fulvestrant therapy to better assess E2 status and its association with treatment efficacy.

Implications for Practice The levels of estradiol in breast cancer women treated with fulvestrant and their potential association with treatment response remain poorly understood. Our findings indicate that estradiol levels exhibit considerable inter-individual variability following fulvestrant treatment. Some women even exhibited premenopausal estradiol levels regardless of whether they received gonadotropin-releasing hormone agonist treatment. Notably, women with elevated estradiol levels who initially responded to fulvestrant experienced significantly worse survival outcomes. Further large-scale studies are warranted to elucidate estradiol levels in this population and, more importantly, the association between estradiol levels and fulvestrant efficacy.

## Introduction

Endocrine therapy is essential for the management of hormone receptor-positive (HR+) breast cancer.^1^ Its efficacy depends on multiple factors, such as prior treatment response and the level of hormone receptor expression.^1^ Suppression of estradiol (E2) is increasingly recognized as a crucial factor, as incomplete E2 suppression in women receiving gonadotropin-releasing hormone agonist (GnRHa)^2^^,^^3^ and/or aromatase inhibitor (AI) therapy^4^^,^^5^ is often associated with reduced treatment efficacy.

An E2 level of 2.72 pg/mL is a widely accepted threshold for adequate suppression^3^^,^^6^^,^^7^ achieved by GnRHa and/or AI therapy, as well as for monitoring treatment response in these women.^8^ Although no association was found between E2 levels and treatment efficacy in tamoxifen-treated women,^9^ long-term tamoxifen use may cause E2 hypersensitivity,^10^ and GnRHa-induced E2 reduction may explain the superior efficacy of tamoxifen plus GnRHa over tamoxifen alone.^11^ These findings suggest that E2 levels are clinically relevant across different types of endocrine therapy.

It remains unclear what the exact E2 levels are in women receiving fulvestrant treatment and whether these levels are associated with treatment efficacy. Fulvestrant,^12^ is now the standard of care when combined with cyclin-dependent kinase (CDK) 4/6 inhibitors for women who have progressed on AI therapy.^13–16^ Its structural similarity to E2 interferes with conventional immunoassays, leading to false-positive results and consequently limiting the availability of reliable data in this population. Liquid chromatography tandem mass spectrometry (LC–MS/MS) is currently the most sensitive and accurate method for evaluating E2 levels, enabling accurate E2 measurement even in the presence of fulvestrant or other agents, thus providing a reliable solution.^17^^,^^18^

Therefore, we used LC–MS/MS to assess E2 levels in women with HR+ advanced breast cancer treated with fulvestrant and further explored the association between E2 levels and clinical outcomes.

## Methods

### Patients’ cohort

This prospective study recruited women with HR+ advanced breast cancer who were receiving fulvestrant therapy and had clinically prescribed E2 testing at Sun Yat-Sen University Cancer Center (SYSUCC). Women enrolled should have been on fulvestrant medication for at least 28 days. Participants who were undergoing active chemotherapy, including antibody–drug conjugate therapy, were excluded. Women receiving trastuzumab, pertuzumab, or pyrotinib for human epidermal growth factor receptor 2 (HER2)-positive status were included, as these agents are not expected to significantly influence E2 levels.^19^

### Ethics statement and patient consent

The study protocol was approved by the institutional review boards of SYSUCC (Ethics approval number B2023-351-01) and was conducted in accordance with the Declaration of Helsinki and the International Conference on Harmonization Good Clinical Practice guidelines. All participants provided written informed consent. The ClinicalTrials.gov number was NCT06195202. This study was observational in nature, and no interventions were administered to the women.

### Study objectives

The primary objective was to determine the proportion of women with HR+ advanced breast cancer on fulvestrant regimens who experienced E2 levels >2.72 pg/mL (10 pmol/L). The threshold of 2.72 pg/mL was selected because it serves as a stringent criterion for postmenopausal status,^7^ which is relevant given that fulvestrant is indicated for use in postmenopausal women. More importantly, it is an established cutoff for inadequate E2 suppression with AI, regardless of concomitant ovarian function suppression (OFS) using GnRHa.^3^^,^^6^^,^^8^ This threshold was applied to all women in our study, regardless of OFS status. Additionally, E2 levels above 10 pg/mL, a widely used cutoff for premenopausal concentrations, were also applied, and the proportion of women exceeding this threshold was classified as ovarian function recovery (OFR).^6^^,^^7^^,^^20^

Secondary objectives included characterizing E2 levels at various time points and comparing progression-free survival (PFS) between women with E2 > 2.72 pg/mL and those with E2 ≤ 2.72 pg/mL. PFS1 was defined as the time interval from the initiation of fulvestrant medication to disease progression (PD) or death. PFS2 was defined as the time interval from blood draw to PD or death. When multiple tests were performed, the peak E2 level was selected, and the time point corresponding to the peak E2 value for each woman was selected to calculate PFS2.

### Hormone assays

Serum samples from women receiving fulvestrant were collected using standard protocols for E2 measurement. Levels were firstly measured by electrochemiluminescence immunoassay (ECLIA) on a Cobas E801 analyzer (Roche Diagnostics, Mannheim, Germany), with an E2 quantification limit of 6 pg/mL.

The samples were stored at −20°C until they were dispatched to Guangzhou KingMed Diagnostics Group Co., Ltd. for E2 analysis.^21^ The quantification of E2 was performed using the TSQ Altis Triple Quadrupole Mass Spectrometer (ThermoFisher Scientific, Waltham, MA). The lower limit of quantification for E2 with LC–MS/MS was determined to be 1.1 pg/mL. Values below 1.1 pg/mL were assigned a value of 1.1 pg/mL.

### Statistical methods

Patient characteristics collected included age, regional recurrence or metastasis, presence of visceral metastasis, prior chemotherapy and endocrine therapy, current use of OFS, CDK 4/6 inhibitors, anti-HER2 targeted therapy, and duration of the current regimen. Patient characteristics were compared for those with E2 levels >2.72 pg/mL vs ≤2.72 pg/mL using Wilcoxon rank sum and Fisher’s exact tests. If multiple tests were conducted, the time point associated with the peak E2 value was selected to calculate the duration of the current regimen.

Time points were categorized into different intervals: <12 months (M, 28 days to 12 months), 12-24 M, and >24 M. One-way analysis of variance (ANOVA) was conducted to compare the median E2 levels across different time points. ­Pearson correlation analysis was employed to assess the correlation between E2 levels measured by ELICA and LC–MS/MS.

Survival analyses were performed using Kaplan–Meier methods, with log-rank tests to compare women with E2 levels >2.72 pg/mL and ≤2.72 pg/mL. Exploratory subgroup survival analyses were conducted exclusively among women who benefited from fulvestrant therapy. These women were defined as those with HR+/HER2− metastatic disease who received first- or second-line fulvestrant therapy for a duration exceeding 6 months, excluding individuals with primary resistance to endocrine therapy. Hazard ratios (HRs) and 95% confidence intervals (CIs) were reported. A post-hoc power analysis was performed to evaluate the statistical power of the subgroup analyses. The sample size was determined by all available data collected during the study period.

The analyses were conducted using Prism 9 version 9.3.1, and statistical significance was defined as *P* < .05.

## Results

### Study population

**Figure 1.**
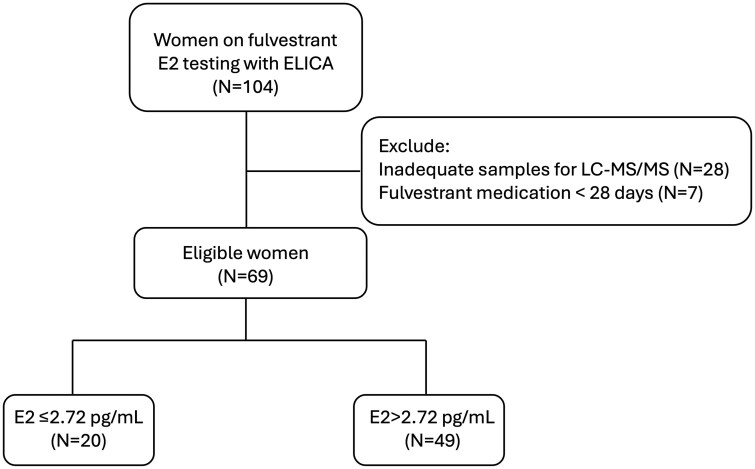
Flow chart of patients’ enrollment.

From January to October 2024, a total of 69 women were recruited for this study, providing 115 blood samples. The patient flow was illustrated in Figure 1.

The baseline characteristics of the participants are summarized in Table 1. The median age of the cohort was 48 years (range: 31-83 years), with 43 women (62.3%) aged under 50 years. A total of 56 women (81.2%) had distant metastasis, and 39 women (56.5%) exhibited visceral metastasis. Fifty women (72.5%) had a history of prior chemotherapy. Three women (4.3%) were diagnosed with HER2-positive disease and were receiving current anti-HER2 targeted therapy. Fifty-one women (73.9%) were undergoing OFS, and 57 women (82.6%) were currently on CDK4/6 inhibitors. Sixty-three women (91.3%) were on first- or second-line fulvestrant therapy. The median duration of the current treatment regimen was 11.6 months (range: 0.9-81.4 months).

**Table 1.** Patients’ characteristics.

Characteristics	*N* (%)			*P*
	E2 > 2.72 pg/mL (*N* = 49)	E2 ≤ 2.72 pg/mL (*N* = 20)	ALL (*N* = 69)	
**Age, years**				.15
** Median (range)**	50 (31-72)	46 (33-83)	48 (31-83)	
**Disease**				.21
** Local/regional recurrence**	11 (22.4)	2 (10)	13 (18.8)	
** Metastasis (recurrence or de novo)**	38 (77.6)	18 (90)	56 (81.2)	
**Distant disease site**				.26
** Visceral**	26 (53.1)	13 (65.0)	39 (56.5)	
** Nonvisceral**	23 (46.9)	7 (35.0)	30 (43.5)	
**Prior therapies**				
** Chemotherapy**	35 (71.4)	15 (75.0)	50 (72.5)	.78
** Endocrine therapy**	35 (71.4)	16 (80.0)	51 (73.9)	.56
**Current therapies**				
** With anti-HER2 targeted therapy**	3 (6.1)	0 (0)	3 (4.3)	.55
** With OFS**	35 (71.4)	16 (80.0)	51 (73.9)	.56
** With CDK4/6 inhibitors**	42 (85.7)	15 (75.0)	57 (82.6)	.31
**Duration of present FUL regimen, months**				.78
** Median (range)**	11.6 (1.4-81.4)	12.0 (0.9-70.0)	11.6 (0.9-81.4)	
**Median prior regimens in the metastatic setting**				.66
** 0-1**	44 (89.8)	19 (95.0)	63 (91.3)	
** ≥2**	5 (10.2)	1 (5.0)	6 (8.7)	

Abbreviations: CDK, cyclin-dependent kinase; E2, estradiol; FUL, fulvestrant; HER2, human epidermal growth factor receptor 2; OFS, ovarian function suppression.

### E2 levels on fulvestrant regimens

The median E2 level for the overall testing samples on fulvestrant regimens (*N* = 115) was 3.60 pg/mL (range: 1.11-526.13 pg/mL) as measured by LC–MS/MS, whereas the median E2 level was 35.74 pg/mL (range: 5.53-635.74 pg/mL) as determined by ELICA. There was a significant positive correlation between fulvestrant levels measured by ELICA and LC–MS/MS (*r* = 0.98, *P* < .0001; Figure S1). The median E2 levels in women with OFS and those without OFS were both 3.6 pg/mL (*P* = .95).

Figure S2 illustrates the distribution of E2 levels and their corresponding time points. Figure S2 shows violin plots of E2 levels at different time points (<12 M, 12-24 M, and >24 M, *N* = 115). The median E2 levels across these time points were not significantly different (*P* = .18, Figure S3), with values of 3.52 pg/mL (<12 M), 3.64 pg/mL (12-24 M), and 3.82 pg/mL (>24 M). Two-thirds (10 out of 15, 66.7%) of premenopausal E2 levels occurred within the first year after initiating the fulvestrant regimen.

### Patients’ characteristics and outcomes according to E2 levels

A total of 49 women (71.0%) exhibited E2 > 2.72 pg/mL. No statistically significant difference was observed between the two cohorts (Table 1). Notably, 11 women (15.9%), accounting for a total of 15 tests, exhibited premenopausal E2 levels >10 pg/mL (OFR). The median age of these women was 47 years (range: 33-52 years), which was younger than that of women without OFR (50 years, *P* = .05). The median duration for premenopausal E2 testing was 6.3 months (range: 1.9-28.0 months), which was shorter than that of women without OFR (13.0 months, *P* = .07). The details were listed in Table S1.

**Figure 2.**
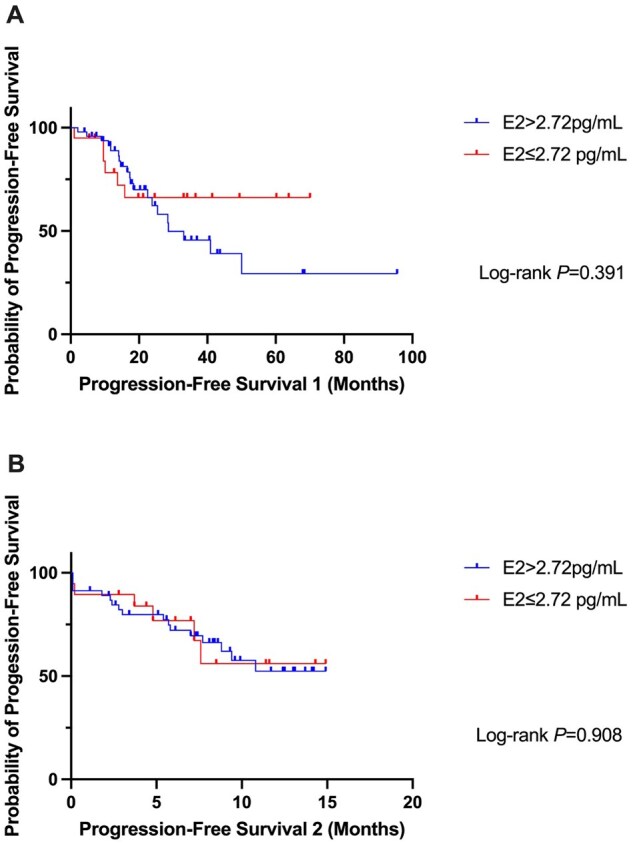
Progression-free survivals (PFS) in all participants. (A) PFS1: from the initiation of fulvestrant medication to disease progression (PD) or death. (B) PFS2: from blood draw to PD or death.

With a median follow-up of 8.4 months (range: 0.6-14.9 months), 27 women had disease progression and 6 women died. Among all participants, events were observed in 20 (40.8%) women with E2 levels >2.72 pg/mL and in 6 (30.0%) women with E2 levels ≤2.72 pg/mL. The median PFS was not reached (NR) in women with E2 ≤ 2.72 pg/mL vs 28.4 months in women with E2 > 2.72 pg/mL (HR = 1.48, *P* = .391, Figure 2A) when evaluating PFS1. Additionally, no statistically significant difference was observed in PFS2 between the two groups (median PFS2 NR in both groups, HR = 1.06, *P* = .908, Figure 2B).

**Figure 3.**
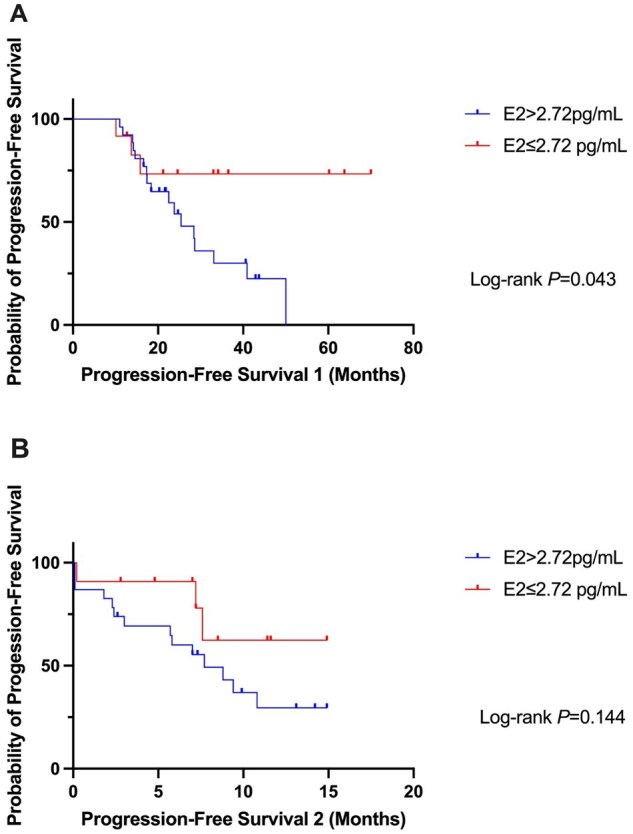
Progression-free survivals (PFS) in patients with metastatic lesions, with first- or second-line fulvestrant regimens >6 months. (A) PFS1: from the initiation of FUL medication to disease progression (PD) or death. (B) PFS2: from blood draw to PD or death.

In the subgroup analysis among the women who benefited from fulvestrant regimens (*N* = 38), those with E2 levels ≤2.72 pg/mL exhibited significantly longer PFS1 compared to those with E2 levels >2.72 pg/mL (NR vs 25.4 months, HR = 3.21, 95% CI, 1.29-8.00, *P* = .043, Figure 3A). PFS2 was longer in the E2 ≤ 2.72 pg/mL group, but the difference was not statistically significant (NR vs 7.7 months, HR = 2.44, *P* = .144, Figure 3B). The patient characteristics were balanced between the two groups (Table S2).

A post-hoc power analysis was performed for the subgroup analyzing PFS1, and the calculated statistical power was approximately 76.1%.

## Discussion

This study is the first to characterize actual E2 concentrations in a cohort of women receiving fulvestrant using LC–MS/MS. Substantial interindividual variability in E2 levels was observed, with an ultra-low median concentration and a certain proportion of women exhibiting premenopausal E2 levels in real-world clinical settings. Whether E2 levels are associated with treatment efficacy warrants further investigation.

Due to the lack of established E2 thresholds for fulvestrant therapy, we used postmenopausal levels as the reference, consistent with its use in postmenopausal women. The E2 cutoff for postmenopausal concentrations ranges from 2.72 pg/mL to 20 pg/mL across various literatures.^6^^,^^7^ Therefore, we chose 2 cutoffs for our analysis: a strict cutoff of 2.72 pg/mL, which is widely used to assess adequate E2 suppression during GnRHa and/or AI treatment,^3^^,^^6^^,^^22^ and a strict threshold corresponding to the postmenopausal stage.^7^ Another cutoff was 10 pg/mL, which was relatively lenient but more commonly used as a threshold for defining the postmenopausal stage.^6^^,^^8^^,^^23^ These two cutoffs represent the most reliable thresholds currently available. Previous studies regarding fulvestrant included few women and consistently showed very low E2 levels.^17^^,^^18^ In our study, interindividual variability in E2 levels ranged from 1.11 pg/mL to 526.13 pg/mL, and 71% of women had E2 levels higher than 2.72 pg/mL. We did not find any factors related to higher E2 levels in this population. Eleven women (15.9%) exhibited premenopausal E2 concentrations. These women were more likely to be younger and to have received a shorter duration of therapy. Two-thirds of the elevated E2 levels occurred within the first 12 months of fulvestrant treatment, suggesting either incomplete OFS or misclassification of menstrual status—findings that are consistent with previous studies in women undergoing GnRHa or AI therapy.^6^^,^^20^^,^^21^ More data regarding women using fulvestrant are needed to further assess the proper E2 levels with a larger sample size.

The relationship between E2 levels and the efficacy of endocrine therapy has garnered significant attention in recent years.^8^^,^^24^ Given the heterogeneity of the overall population, no statistically significant difference in PFS was observed between the groups with E2 > 2.72 pg/mL and those with E2 ≤ 2.72 pg/mL. However, a significant association was observed between higher E2 levels and shorter PFS in HR+/HER2- women receiving long-term fulvestrant regimens (HR: 3.21). A post-hoc power analysis showed the statistical power was 76.1%. Although the subgroup analysis was underpowered due to a small sample size, the observed significance underscores the strength of the association and supports larger studies for confirmation. Some adverse impact on survival likely stems from OFR, given its established role in compromising the efficacy of AI and OFS.^2^^,^^3^^,^^20^ However, this is unlikely to affect overall outcome measures, as OFR is a recognized phenomenon in both clinical research^6^^,^^20^ and real-world practice.^21–23^ Numerous studies have retrospectively examined the potential impact of OFR on patient outcomes.^2^^,^^4^^,^^20^ Due to the limited sample size, we were not able to evaluate the efficacy of fulvestrant in relation to OFR. Based on the above considerations, we hypothesize that incorporating E2 monitoring in women receiving fulvestrant therapy will enable a more comprehensive and clinically meaningful assessment of E2 throughout the course of treatment.

The positive correlation between PFS and suppressed E2 observed in the subgroup analysis aligns with previous literature. In Berstein’s study,^10^ long-term tamoxifen exposure increases sensitivity to E2 in breast cancer, suggesting the E2 suppression may be crucial. This hypothesis was supported by the SOFT study, which demonstrated that the combination of GnRHa and tamoxifen provides a survival benefit over tamoxifen monotherapy.^11^ In our subgroup analysis, patients exhibiting primary resistance (PFS < 6 months) were excluded first. In women who respond to fulvestrant, we hypothesize that E2 sensitivity may increase during long-term therapy, leading to elevated E2 levels that promote tumor progression and poorer survival outcomes. In addition, elevated E2 levels were predominantly observed during the first year of therapy but remained stably suppressed thereafter. This suggests that only women with adequate suppression can continue fulvestrant long-term, while those with persistently elevated levels may experience early disease progression.

Although E2 levels were measured at varying time points rather than at fixed intervals in our study, we assume that this variability is unlikely to have affected the study results. First, standardized timing for E2 measurement was not established. The median time to E2 testing in our study was 11.6 months, which is sufficient to assess hormonal stability during treatment. Finally, women who received fulvestrant for less than 28 days were excluded to ensure stable E2 levels.

The limitations of our study were as follows: First, the limited sample size may have compromised the statistical power. Second, the E2 threshold of 2.72 pg/mL for fulvestrant, derived from AI data, might not be optimal; however, it remains the most appropriate cutoff currently available for clinical application. Third, the survival analysis was exploratory and should be interpreted with caution. Nevertheless, as previously mentioned, the HR and its confidence interval indicate a statistically significant association.

In conclusion, we describe the inter-individual variability in E2 levels among women receiving fulvestrant. A certain proportion of women were in premenopausal E2 levels. There was a significant association between higher E2 levels and poorer PFS in women receiving long-term fulvestrant therapy. Our findings highlight the critical need for monitoring E2 levels in women receiving fulvestrant therapy to better understand its true concentrations and the association between E2 levels and treatment efficacy.

## Supplementary Material

oyaf403_Supplementary_Data

## Data Availability

The datasets used and/or analyzed during the current study are available from the corresponding author on reasonable request.
