# Supplementary material for: Estradiol levels in women with hormone receptor-positive advanced breast cancer on fulvestrant therapy
Source: Oncologist. 2025 Dec 5;30(12):oyaf403. doi: 10.1093/oncolo/oyaf403 (PMC12715405; doi:10.1093/oncolo/oyaf403)
Supplement: oyaf403_Supplementary_Data [file oyaf403_supplementary_data.zip › Supplementary_File/Table S1. OFR Patients characteristics .docx]

| Characteristics | N (%) | | *P* |
| --- | --- | --- | --- |
|  | E2 > 10 pg/mL  (N = 11) | E2 ≤ 10 pg/mL  (N = 58) |  |
| Age, years | | | |
| Median (Range) | 47 (33-51) | 50 (31-83) | 0.05 |
| Disease | | | 0.34 |
| Local/Regional Recurrence | 3 (27.3) | 10 (17.2) |  |
| Metastasis (Recurrence or De novo) | 8 (72.7) | 48 (82.8) |  |
| Distant Disease site | | | 0.32 |
| Visceral | 5 (45.5) | 34 (58.6) |  |
| Nonvisceral | 6 (54.5) | 24 (41.4) |  |
| Prior therapies | | | |
| Chemotherapy | 9 (81.8) | 41 (85.4) | 0.36 |
| Endocrine therapy | 10 (90.9) | 41 (85.4) | 0.15 |
| Current therapies |  |  |  |
| With OFS | 9 (81.8) | 42 (87.5) | 0.41 |
| With CDK4/6 inhibitors | 8 (72.7) | 49 (84.5) | 0.29 |
| Duration of present fulvestrant regimen, months | | | 0.07 |
| Median (Range) | 6.3 (1.9-28.0) | 13.0 (0.9-81.0) |  |
| Median prior regimens in the metastatic setting | | | 0.24 |
| 0-1 | 9 (81.8) | 54 (93.1) |  |
| ≥2 | 2 (18.2) | 4 (6.9) |  |

E2, estradiol; HER2, human epidermal growth factor receptor 2; OFS, ovarian function suppression; CDK, cyclin-dependent kinase
