# Supplementary material for: Estradiol levels in women with hormone receptor-positive advanced breast cancer on fulvestrant therapy
Source: Oncologist. 2025 Dec 5;30(12):oyaf403. doi: 10.1093/oncolo/oyaf403 (PMC12715405; doi:10.1093/oncolo/oyaf403)
Supplement: oyaf403_Supplementary_Data [file oyaf403_supplementary_data.zip › Supplementary_File/Table S2. PFS Patients characteristics .docx]

| Characteristics | N (%) | | | *P* |
| --- | --- | --- | --- | --- |
|  | E2 > 2.72 pg/mL  (N = 26) | E2 ≤ 2.72 pg/mL  (N = 12) | ALL  (N = 38) |  |
| Age, years | | | | 0.48 |
| Median (Range) | 50 (31-59) | 46 (33-83) | 48 (31-83) |  |
| Disease | | | |  |
| Metastasis (Recurrence or De novo) | 26 (100) | 12 (100) | 38 (100) |  |
| Distant Disease site | | | | 0.12 |
| Visceral | 15 (57.7) | 10 (83.3) | 25 (65.8) |  |
| Nonvisceral | 11 (42.3) | 2 (16.7) | 13 (34.2) |  |
| Prior therapies | | | | |
| Chemotherapy | 21 (80.8) | 8 (66.7) | 29 (76.3) | 0.29 |
| Endocrine therapy | 20 (76.9) | 9 (75.0) | 29 (76.3) | 0.60 |
| Current therapies |  |  |  |  |
| With OFS | 23 (88.5) | 9 (75.0) | 32 (84.2) | 0.27 |
| With CDK4/6 inhibitors | 24 (92.3) | 10 (83.3) | 34 (89.5) | 0.38 |
| Duration of present FUL regimen, months | | | | 0.65 |
| Median (Range) | 17.0 (6.1-44.0) | 19.0 (6.1-70.0) | 16.9 (6.1-70.0) |  |
| Median prior regimens in the metastatic setting | | | |  |
| 0-1 | 26 (100) | 12 (100) | 38 (100) |  |

E2, estradiol; HER2, human epidermal growth factor receptor 2; OFS, ovarian function suppression; CDK, cyclin-dependent kinase; FUL, fulvestrant.
