# Supplementary figures and images for: Estradiol levels in women with hormone receptor-positive advanced breast cancer on fulvestrant therapy
Source: Oncologist. 2025 Dec 5;30(12):oyaf403. doi: 10.1093/oncolo/oyaf403 (PMC12715405; doi:10.1093/oncolo/oyaf403)

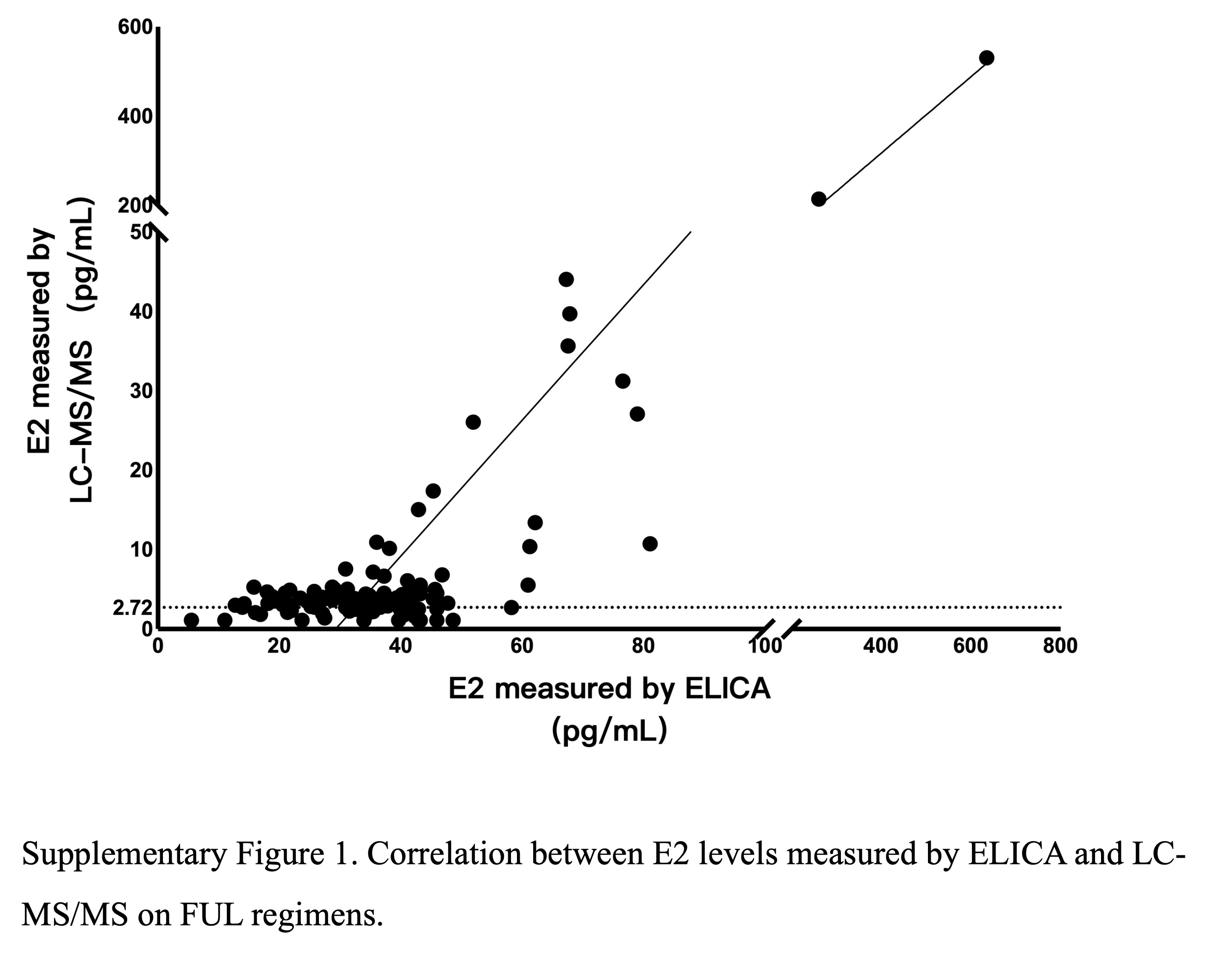

Supplement: oyaf403_Supplementary_Data [file oyaf403_supplementary_data.zip › Supplementary_File/Figure S1 1112.jpg]

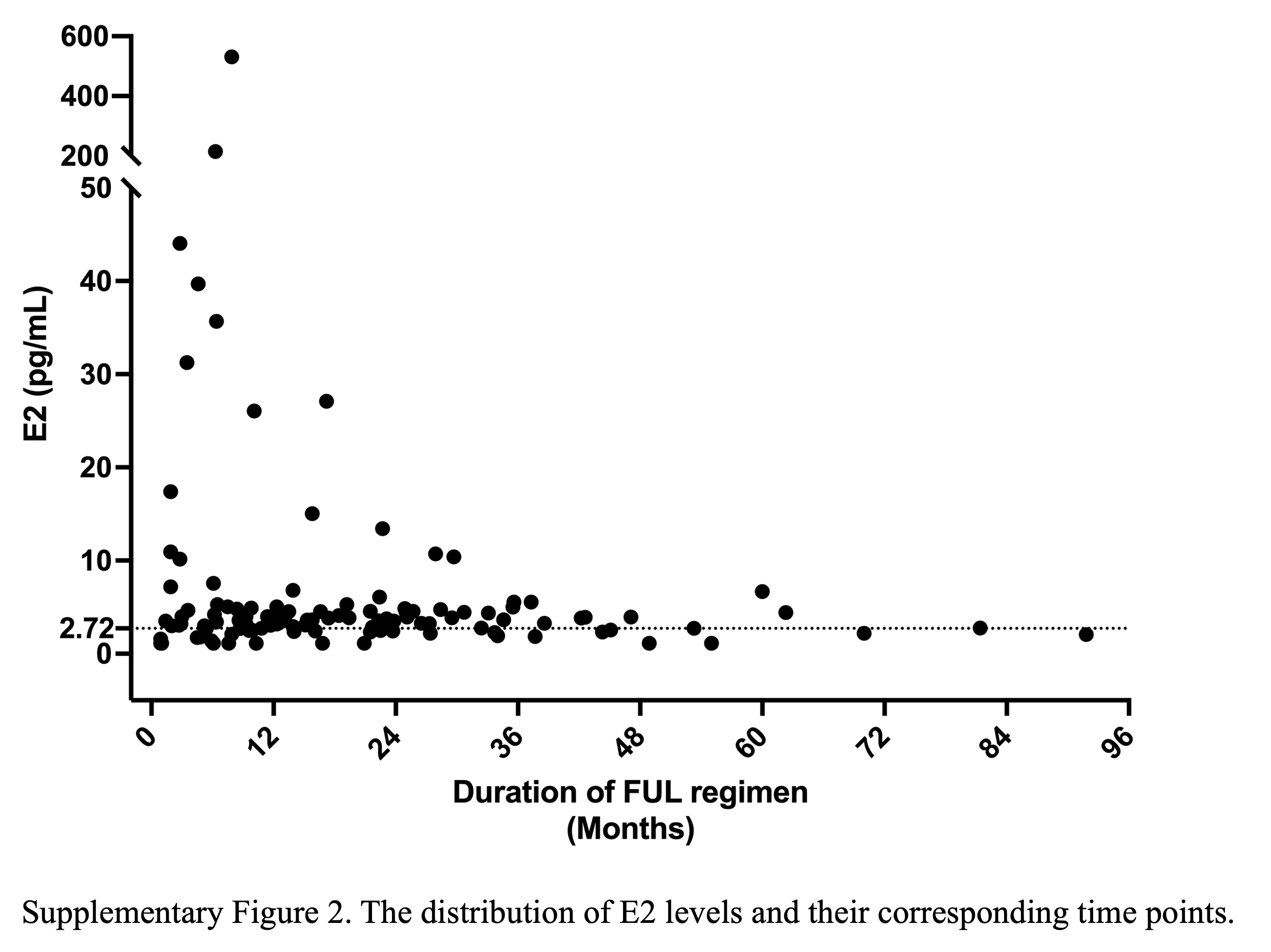

Supplement: oyaf403_Supplementary_Data [file oyaf403_supplementary_data.zip › Supplementary_File/Figure S2 1112.jpg]

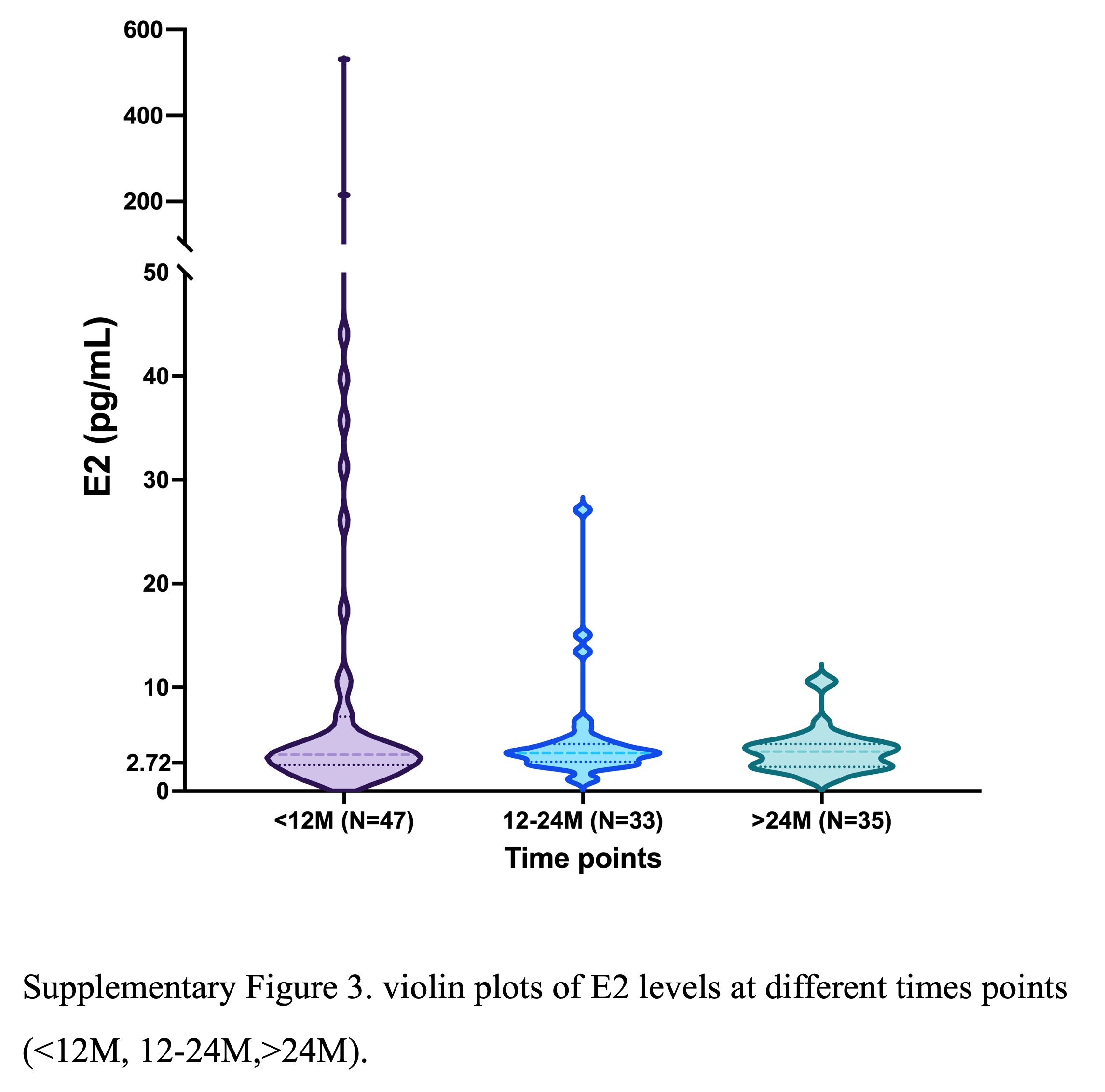

Supplement: oyaf403_Supplementary_Data [file oyaf403_supplementary_data.zip › Supplementary_File/Figure S3 1112.jpg]
